# Outcomes and Costs of Treating Hepatitis C Patients in the Era of First Generation Protease Inhibitors – Results from the PAN Study

**DOI:** 10.1371/journal.pone.0159976

**Published:** 2016-07-28

**Authors:** Jona T. Stahmeyer, Siegbert Rossol, Florian Bert, Klaus H. W. Böker, Harald-Robert Bruch, Christoph Eisenbach, Ralph Link, Christine John, Stefan Mauss, Renate Heyne, Eckart Schott, Heike Pfeiffer-Vornkahl, Dietrich Hüppe, Christian Krauth

**Affiliations:** 1 Institute for Epidemiology, Social Medicine and Health Systems Research, Hannover Medical School, Hannover, Germany; 2 Department of Internal Medicine, Krankenhaus Nordwest, Frankfurt a.M., Germany; 3 Leberpraxis, Hannover, Germany; 4 Schwerpunktpraxis Bonn, Bonn, Germany; 5 University Hospital Heidelberg, Dept. of Gastroenterology, Heidelberg, Germany; 6 St. Josefs Hospital, Offenburg, Germany; 7 Medical Practice, Berlin, Germany; 8 Center for HIV and Hepatogastroenterology, Duesseldorf, Germany; 9 Leberzentrum am Checkpoint, Berlin, Germany; 10 Dept. of Hepatology and Gastroenterology, Charité Universitätsmedizin Berlin, Campus Virchow Klinikum, Berlin, Germany; 11 e-factum – GmbH, Butzbach, Germany; 12 Center of Gastroenterology, Herne, Germany; University of North Carolina School of Medicine, UNITED STATES

## Abstract

**1. Objective:**

Chronic hepatitis C virus infections (HCV) cause a significant public health burden. Introduction of telaprevir (TVR) and boceprevir (BOC) has increased sustained virologic response rates (SVR) in genotype 1 patients but were accompanied by higher treatment costs and more side effects. Aim of the study was to assess outcomes and costs of treating HCV with TVR or BOC in routine care.

**2. Material and Methods:**

Data was obtained from a non-interventional study. This analysis relates on a subset of 1,786 patients for whom resource utilisation was documented. Sociodemografic and clinical parameters as well as resource utilisation were collected using a web-based data recording system. Costs were calculated using official remuneration schemes.

**3. Results:**

Mean age of patients was 49.2 years, 58.6% were male. In treatment-naive patients SVR-rates of 62.2% and 55.7% for TVR and BOC were observed (prior relapser: 68.5% for TVR and 63.5% for BOC; prior non-responder: 45.6% for TVR and 39.1% for BOC). Treatment costs are dominated by costs for pharmaceuticals and range between €39,081 and €53,491. We calculated average costs per SVR of €81,347 (TVR) and €70,163 (BOC) in treatment-naive patients (prior relapser: 78,089 €/SVR for TVR and 82,077 €/SVR for BOC; prior non-responder: 116,509 €/SVR for TVR and 110,156 €/SVR for BOC). Quality of life data showed a considerable decrease during treatment.

**4. Conclusion:**

Our study is one of few investigating both, outcomes and costs, of treating HCV in a real-life setting. Data can serve as a reference in the discussion of increasing costs in recently introduced agents.

## Introduction

Chronic hepatitis C virus infections (HCV) cause a significant public health burden. Worldwide, about 170 million people are chronically infected with HCV [1]. Data from the German National Health and Examination Survey (DEGS1) estimate an anti HCV-prevalence of 0.3% in the general population [2]. More extended studies which consider a higher prevalence in risk-groups such as drug abusers, prison inmates and some urban areas, estimated the number of patients with a viremic infection at 275,000 (165,000–494,000) in Germany [3;4]. The majority of patients have acquired genotype 1 or 3 hepatitis C virus [5]. A large part of infected patients are unaware of their disease. Most infections remain undiagnosed until serious and potentially lethal complications such as liver cirrhosis and hepatocellular carcinoma occur [6]. Estimates assume that about 27% of liver cirrhosis and 25% of hepatocellular carcinoma (HCC) are attributable to chronic HCV [7]. Further, chronic hepatitis C is still the most common cause for liver transplantations [8]. The primary goal of treating hepatitis C is to eradicate the virus (sustained viral response, SVR) and thus to reduce the morbidity and mortality of infected patients. Recent studies have shown that SVR is associated with a decreasing risk of liver cirrhosis, liver-related mortality and all-cause mortality as well as an increase in quality of life [9–11].

The introduction of first generation protease inhibitors telaprevir (TVR) and boceprevir (BOC) for the treatment of patients infected with HCV genotype 1 in 2011 has significantly increased SVR rates while shortening treatment duration for many patients. Especially treatment-experienced patients who failed to previous treatment attempts with peginterferon (PegIFN) and ribavirin (RBV) dual therapy have benefited from these treatment options. In 2012, the Joint Federal Committee (Gemeinsamer Bundesausschuss) estimated the number of patients eligible for treatment at 46,000 in Germany (treatment-naive: 12,000; treatment-experienced 34,000) [12;13]. From 2011 to 2014, triple therapy with TVR or BOC has been established as the new standard of care [14]. However, triple-therapy with first generation protease inhibitors is associated with significantly increased adverse events and patients with advanced fibrosis or other comorbidities are often not eligible for treatment [15]. Further, treatment options are associated with increased costs. Although several cost-effectiveness studies have shown that treatment with TVR or BOC is cost-effective, there is still a lack of studies evaluating outcomes and costs of treating hepatitis C with TVR and BOC in a real-life setting.

A study by Bichoupan et al. (2014) showed extremely high costs of $189,000 per SVR for patients receiving first generation protease inhibitors [16]. In another real-life study average SVR-rates of 53% for TVR and 40% for BOC were observed [17]. Due to small sample sizes, extensive subgroup analyses could not be conducted and generalisation of these results is doubtful. First interim data from the “**P**eginterferon **a**lfa-2a **n**on-interventional trial” (PAN) cohort showed SVR-rates in treatment-naive patients of 63.4% and 55.0% for TVR and BOC, respectively. Results were lower in treatment-experienced patients, except for patients with prior relapse [18].

Therefore, the aim of this study was to analyse outcomes and healthcare costs of treating patients with TVR or BOC in routine care using the largest cohort of HCV infected patients so far. Further, information on quality of life was collected.

## Methods

The data analyzed are part of the PAN trial. The PAN study is a prospective, open-label, multicenter, non-interventional study started in 2011 and initiated by the Association of German Gastroenterologists in Private Practice (Berufsverband Niedergelassener Gastroenterologen Deutschlands e.V.) in cooperation with Roche Pharma AG, Germany. It is a continuation of a non-interventional study analyzing effectiveness and treatment costs of patients receiving peginterferon and ribavirin [19–21]. The design of the study was approved by the Ethical Committee of the Aerztekammer Westfalen-Lippe and patients gave written informed consent before taking part in the study. Inclusion criteria were indication for therapy of chronic hepatitis C according to the attending physician as well as an age of 18 years or above. Federal health authorities were infomed about the study: The trial was registered at the Association of Researching Pharmaceutical Manufacturers (ML25724) and at ClinicalTrials.gov (NCT01679834). Sponsor according to GCP/GEP of the non-interventional study was Roche Pharma AG, Germany. The authors were independent from the funding institutions in data analysis, data interpretation, report writing and publication.

A web-based data recording system was used for data collection. The quality of the data was assessed by automated plausibility checks as well as additional on-site monitoring. Data collection included treatment outcomes (SVR), sociodemographic and clinical parameters, HCV-associated health care utilisation and quality of life (QoL). SVR was defined as undetectable HCV RNA at least 10 weeks after end of treatment (EoT). This definition was used because timing of patient visits was not specified and thus some patients may not have had a physician visit at exactly 12 or 24 weeks after end of treatment. Accordingly, patients without HCV RNA determination 10 weeks or later after EoT or patients who were lost to follow-up were considered to have failed treatment in our analysis (intention-to-treat analysis).

Healthcare utilisation comprises outpatient care (doctor visits, laboratory testing and imaging), pharmaceuticals (antiviral treatment and medication for the treatment of HCV-associated diseases or treatment-related adverse events) as well as HCV-associated hospitalisation. Costs of outpatient care and services are based on the German doctors’ fee scale within the statutory health insurance scheme (Einheitlicher Bewertungsmaßstab), pharmaceutical costs were calculated using prices from the German drug directory (Rote-Liste) and hospital costs were calculated based on the German hospital reimbursement scheme (G-DRG) [22–24]. Price year was 2012.

Information on QoL was collected using the German version and transformation of the Short-Form-36 version 2 (SF-36v2). Patients were asked to fill out the questionnaires at four different times of treatment: (1.) baseline, (2.) treatment week 12, (3.) end of treatment, (4.) 10 to 24 weeks post treatment (SVR evaluation).

The present analysis is based on data from a defined subset of HCV-patients receiving antiviral treatment with TVR or BOC in combination with PegIFN and RBV and for whom detailed information on resource utilisation was documented. Patients with HIV co-infection and/or patients reveiving drug substitution treatment were excluded. Treatment was recommended to be in accordance with the drug safety: SmPC (summary of product characteristics) and national treatment guidelines but was ultimately at the discretion of the physician in charge. In total, data on 1,786 genotype 1 patients were analysed. Data were gathered between June 2011 and January 2014 and provided by 191 participating centers (160 outpatient practices and medical centers as well as 31 hospital-based outpatient clinics) all throughout Germany and thus reflects the accepted practice of HCV-treatment in Germany.

Statistical analyses are primarily descriptive to reflect the non-interventional character of the study. We used SPSS Statistics 12 for the analyses. Differences between subgroups were analysed using the T-Test or χ²-Test depending in type of variable. Differences were regarded as significant at a level of p≤0.05.

## Results

### Patient Characteristics

Overall, data on 1,786 patients receiving triple-therapy with BOC (25.3%) or TVR (74.7%) were analysed. Mean age was 49.2 years (SD 11.2), 58.6% were male. Mean height and weight were 172.3 cm (SD 9.4) and 79.2 kg (SD 15.3), respectively (Body-Mass-Index 26.6; SD 4.5). Almost all patients were Caucasian. Estimated duration of infection was 16.9 years (SD 10.9) on average. Most common routes of transmission were historical intravenous drug abuse (19.4%), blood products (17.4%) and surgical/medical interventions (8.5%). For almost half of the patients (49.7%) the route of transmission was unknown. About half of patients were treatment-naive (44.5%), 29.8% had a relapse and 22.6% had non-response to previous therapy with PegIFN/RBV. Other patients (2.8%) discontinued previous treatment due to adverse events and/or personal reasons and six patients (0.3%) had a reinfection. Comorbidities were frequent in our sample (58.9%). Most frequent diseases were cardiovascular diseases (20.5%), psychiatric disorders (12.4%), thyroidal dysfunction (9.1%) and diabetes mellitus (7.8%). 15 patients (0.8%) were co-infected with hepatitis B virus. A history of alcohol and/or drug abuse was observed for 18.4% of patients.

Baseline viral load was available for 1,742 patients (97.5%). Average viral load was 3.01 million IU; 27.2% of patients had a low viral load (≤400,000 IU/ml) and 72.8% had a high viral load (>400,000 IU/ml). Mean levels of AST and ALT were 67.7 U/I and 92.7 U/I, respectively.

Due to the non-interventional character of the study, no standardized evaluation on liver fibrosis was performed. Information on liver fibrosis/cirrhosis was gathered by available information. Most patients (79.9%) received at least one ultrasound examination of the liver; 15.8% had a liver biopsy. Information on other methods for evaluation of liver fibrosis (e.g. FibroScan, FibroTest or acoustic radiation force impulse) was not sufficiently documented. Further, these methods are not covered by the statutory health insurance and have to be paid by the patients themselves. Based on available data (e.g. biopsy, clinical appearance, sonography, elastography) 14.2% of patients were classified as cirrhotics. We further used APRI-score to determine the degree of liver fibrosis/cirrhosis. Mean APRI-score was 1.0 (SD 1.32; n = 1,483). An APRI-score of ≤0.5 indicating no significant fibrosis was observed in 42.9% of patients, 39.8% had a score >0.5 and ≤1.5 and 17.3% had a score >1.5 indicating advanced fibrosis or liver cirrhosis. Patient characteristics are summarized in Table 1.

**Table 1:** Patient characteristics (n = 1,786, otherwise stated).

Parameter	All patients (n = 1,786)	TVR	BOC	TVR vs. BOC p-value
Age, years	49.2	49.3	49.0	n.s.
Sex, %				
male	58.6	60.7	52.1	0.0013
female	41.4	39.3	47.9	
Height, cm	172.3	172.5	171.7	0.0063
Weight, kg	79.2	79.0	79.8	n.s.
Body-Mass-Index	26.6	26.5	27.0	0.0397
Duration of infection, years	16.9	16.6	17.8	0.0425
Medical history				
Treatment-naive	44.5	41.0	54.5	<0.001
Relapser	29.8	32.1	23.1	
Non-responder	22.6	23.7	19.3	
Other (incl. re-infection)	3.1	3.2	3.1	
Route of transmission, %				
Drug abuse	19.4	19.0	20.6	n.s.
Blood products	17.4	17.7	16.6	
Medical/surgical intervention	8.5	7.9	10.0	
Sexual contact	1.7	1.3	2.7	
other	3.4	3.3	3.8	
unknown	49.7	50.8	46.3	
Frequent comorbidities, %				
Cardiovascular disease	20.5	21.0	19.1	n.s.
Psychiatric disorder	12.4	11.8	14.2	n.s.
Thyroidal dysfunction	9.1	9.3	8.4	n.s.
Diabetes mellitus	7.8	8.1	6.9	n.s.
History of drug/alcohol abuse	18.4	17.5	20.8	n.s.
HBV-coinfection	0.8	0.7	1.1	n.s.
Baseline viral load, IU/ml	3,0 x 10^6^	3,0 x 10^6^	3,1 x 10^6^	n.s.
Baseline viral load, % (n = 1,742)				
≤ 400,000 IU/ml	27.2	26.7	28.6	n.s.
> 400,000 IU/ml	72.8	73.3	71.4	
AST, U/l	67.7	69.4	62.8	0.0106
ALT, U/l	92.7	95.5	84.5	n.s.
APRI-score at baseline (n = 1,483)	1.0	1.1	0.9	0.0122
APRI-score at baseline categorized, % (n = 1,483)				
≤ 0.5	42.9	42.3	44.8	n.s.
> 0.5 and ≤ 1.5	39.8	39.2	41.5	
> 1.5	17.3	18.5	13.7	

### Treatment response

Overall, we observed an SVR-rate of 58.2% in our total sample who received treatment with either TVR or BOC (n = 1,786). In treatment-naive patients SVR-rates of 62.2% and 55.7% were observed for TVR and BOC, respectively. Difference between TVR and BOC treatment was not significant (p = 0.085, mean difference 6.5%, 95%-CI -0.9–14.0). SVR-rates in patients with relapse to previous treatment were 68.5% in patients receiving TVR and 63.5% in patients receiving BOC (p = 0.35, mean difference 5.0%, 95%-CI -5.3–15.4). In patients with non-response to previous treatment SVR-rates were 45.6% in TVR and 39.1% in BOC (p = 0.33, mean difference 6.5%, 95%-CI -5.3–18.3).

In total, 476 of 1,786 patients (26.7%) discontinued treatment with BOC or TVR. Reasons for treatment discontinuation were insufficient virologic response, adverse events or other non-medical reasons.

Average treatment duration in treatment-naive patients was 27.2 weeks in TVR and 31.7 weeks in BOC. 49.8% of treatment-naive patients received TVR for about 24 weeks and 18.8% for about 48 weeks. In patients receiving BOC 52.0% received treatment for about 28 weeks and 24.0% for 48 weeks. In treatment-experienced patients treatment durations were 31.6 weeks (38.5% received treatment for about 24 weeks and 35.7% for about 48 weeks) and 41.2 weeks (11.5% received treatment for about 28 weeks and 70.2% for about 48 weeks) in patients with prior relapse and 33.0 weeks (11.4% received treatment for about 24 weeks and 45.8% for about 48 weeks) and 34.7 weeks (10.3% received treatment for about 28 weeks and 51.7% for about 48 weeks) in patients with prior non-response for TVR and BOC, respectively. Information on response rates and treatment duration are summarized in Table 2. Shorter treatment duration in BOC patients with prior non-response compared to prior relapsers is due to more frequent treatment discontinuations.

**Table 2:** Treatment outcomes by patient group.

			*Treatment outcome*		
Patient group	n	Treatment duration, weeks	*SVR*, *%*	*No SVR*, *% (Patients with treatment discontinuation due to non-response or AEs or personal reasons and patients with relapse)*	*Lost to follow-up*, *not tested*, *%*
**Treatment-naive**	**794**	**28.6**	**60.2**	**29.3**	**10.5**
TVR	548	27.2	62.2	29.2	8.6
BOC	246	31.7	55.7	29.7	14.6
**Treatment-experienced**					
**Prior Relapser**	**533**	**33.5**	**67.5**	**25.4**	**7.1**
TVR	429	31.6	68.5	25.0	6.5
BOC	104	41.2	63.5	25.9	9.6
**Prior Non-Responder**	**403**	**33.3**	**44.2**	**49.3**	**6.5**
TVR	316	33.0	45.6	48.1	6.3
BOC	87	34.7	39.1	54.0	6.9

### Adverse events

Adverse events (AE) were common in patients receiving TVR or BOC treatment. 90.5% of patients had at least one non-serious AE. Most common non-serious AEs were fatigue (62.9%), skin diseases (35.9%), pruritus (31.2%), nausea (25.8%), anemia (25.6%), headache (22.3%), arthralgia (17.7%), sleeplessness (16.2%), depressive mood (14.4%) and fever (13.4%).

Serious adverse events were observed in 11.2% of treated patients. Most frequent SAEs were anemia (42 patients; 2.4%), rash (14 patients; 0.8%), pancytopenia (10 patients; 0.6%), fever (7 patients, 0.4%) and sepsis (7 patients, 0.4%). 4.3%% (TVR) and 0.9% (BOC) of patients discontinued therapy due to adverse events.

### Resource utilisation and costs

On average, patients had 12.1 doctor visits. However, patients receiving treatment with TVR had less doctor visits compared to BOC patients (11.7 vs. 13.3; p<0.0001). Besides antiviral treatment with either TVR or BOC, nearly all patients (93.5%) received co-medication for the treatment of HCV-associated diseases or therapy related side effects. 100 patients (5.6%) needed to be treated as inpatients due to their HCV-infection or therapy related side effects. Average length of inpatient care was 4.8 days.

Calculation of treatment costs is based on 1,784 patients as complete resource utilisation data were not available for two patients (one TVR and one BOC). The costs for ambulatory care amount for €1,098 on average (TVR: €1,071; BOC: €1,179). These include costs for doctor visits, laboratory services and imaging techniques which are necessary for initiating antiviral treatment as well as treatment monitoring. Average pharmaceutical costs were €48,562 and contain antiviral treatment with TVR or BOC with PegIFN and RBV (€48,549) and costs for co-medication (€13). Antiviral treatment costs were higher in patients receiving triple-therapy with TVR (€50,862) compared to BOC patients (€41,693). Average costs for co-medication were almost identical (TVR: €13; BOC: €14). Mean costs for inpatient care were €165 for the total population (TVR: €188; BOC: €99). Average costs in 100 patients who were treated as inpatients were €2,946 (TVR: €2,979; BOC: €2,778). Total treatment costs sum up for €49,826 (TVR: €52,120; BOC: €42,970). Further, costs were stratified by treatment status (Table 3).

**Table 3:** Treatment costs by patient group.

			*Costs*, *€*			
Patient group	n	Number of doctor visits	*Ambulatory care*	*Pharmaceuticals*	*Inpatient care*	*Total*
**Treatment-naive**						
TVR	548	11.2	1,026	49,439	133	50,598
BOC	246	12.5	1,119	37,843	119	39,081
**Treatment-experienced**						
**Prior Relapser**						
TVR	429	11.7	1,087	52,150	254	53,491
BOC	103	15.3	1,334	50,766	119	52,119
**Prior Non-Responder**						
TVR	316	12.8	1,123	51,804	201	53,128
BOC	87	13.8	1,171	41,866	34	43,071

### Costs per SVR

Based on SVR-rates and cost estimates in different patient groups we determined how much money has to be spent to achieve SVR in one person with HCV. In treatment-naive patients average costs were 81,347 €/SVR for patients receiving TVR and 70,163 €/SVR for patients treated with BOC. Average costs per SVR in patients with relapse to prior treatment were €78,089 and €82,077 for TVR and BOC, respectively. In patients with non-response to previous treatment average costs were 116,509 €/SVR (TVR) and 110,156 €/SVR (BOC). Costs per SVR estimates are summarized in Table 4.

**Table 4:** Costs per SVR for patient groups.

Patient group	n	SVR, %	Treatment costs, €	Costs per SVR, €
**Treatment-naive**				
TVR	548	62.2	50,598	81,347
BOC	246	55.7	39,081	70,163
**Treatment-experienced**				
**Prior Relapser**				
TVR	429	68.5	53,491	78,089
BOC	103	63.5	52,119	82,077
**Prior Non-Responder**				
TVR	316	45.6	53,128	116,509
BOC	87	39.1	43,071	110,156

### Quality of life

Complete information on quality of life was not available for the total study population as some patients did not fill out the questionnaires at all or did not fill out questionnaires regularly. Therefore, we used three different sub-samples to conduct quality of life analyses and to use all patients available:

1. Quality of life of infected patients (baseline analysis)
2. Development of quality of life during treatment (complete data analysis)
3. Differences in quality of life depending on SVR (10 weeks or later post treatment analysis)

Complete data on QoL at baseline were available for 422 patients. The analysis supports the evidence that chronic HCV infection is associated with reduced QoL compared to QoL in the general population. Biggest QoL restrictions were observed for general health (-19%), vitality (-19%) and role-emotional (-15%). All SF-36 QoL subscales showed significantly lower values, except for bodily pain. Further, QoL sumscores were significantly lower compared to general population (Table 5).

**Table 5:** Quality of life at baseline.

	HCV-patients			German norm population			
SF-36 scales	Mean	SD	n	Mean	SD	Diff	p-value
Physical functioning	81	22.8	432	86	22.3	4.6	<0.0001
Role—physical	73	38.2	431	83	32.6	10.0	<0.0001
Bodily pain	80	25.7	433	79	27.4	-0.9	0.5107
General health	57	19.9	430	68	20.3	10.9	<0.0001
Vitality	53	21.8	430	63	18.5	10.2	<0.0001
Social functioning	79	22.8	433	89	18.4	9.8	<0.0001
Role—emotional	78	37.2	427	90	26.3	12.0	<0.0001
Mental health	68	18.5	430	74	16.6	5.8	<0.0001
**Sumscores:**							
Physical component summary score	49	9.1	422	50	10.3	1.1	0.0459
Mental component summary score	44	12.3	422	51	8.2	7.4	0.0001

**Fig 1:**
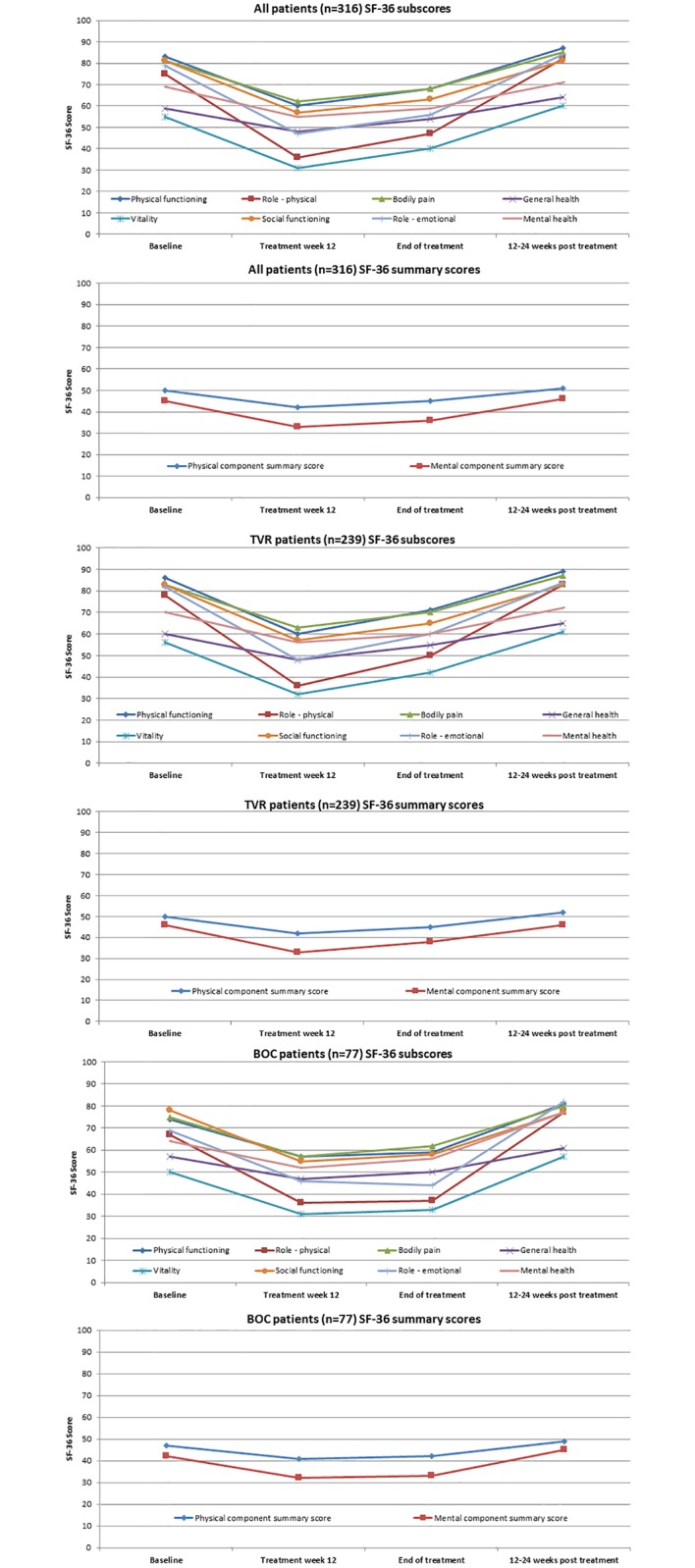
QoL development during treatment.

To analyse the reduction as well as the development of quality of life during antiviral treatment all patients who participated in all surveys (baseline, treatment week 12, end of treatment, 24 weeks post treatment) were analyzed (n = 316). The analysis shows a significant decrease in quality of life during treatment (baseline vs. treatment week 12) for all SF-36 scales, whereas decrease is greatest in role-physical (-52.0%), vitality (-43.6%) and role-emotional (-40.5%). Development of quality of life is described in Fig 1. At the end of treatment QoL increases. Detailed information on QoL stratified for TVR and BOC is presented in the appendix. Post treatment QoL scales show higher or equal values compared to baseline. A comparison of patients who achieved SVR and patients who failed treatment demonstrates that achievement of SVR is associated with an increased QoL. All SF-36 subscales, except role-physical and bodily pain, show higher values (Table 6).

**Table 6:** Post treatment QoL—SVR vs. no SVR.

	No SVR (n = 106)		SVR (n = 378)		No SVR vs. SVR	Total (n = 484)	
SF-36 scales	Mean	SD	Mean	SD	p-value	Mean	SD
Physical functioning	81	20.7	86	21.4	0.0328	85	21.3
Role—physical	76	37.4	82	34.2	0.1187	80	34.9
Bodily pain	81	23.4	85	23.1	0.1168	84	23.2
General health	54	20.8	67	20.5	0.0001	64	21.2
Vitality	53	20.8	63	20.4	0.0001	61	20.8
Social functioning	75	24.6	83	21.9	0.0013	81	22.7
Role—emotional	75	41.4	84	33.8	0.0218	82	35.7
Mental health	66	18.7	72	18.7	0.0037	71	18.9
**Sumscores:**							
Physical component summary score	49	8.6	51	8.2	0.0286	51	8.3
Mental component summary score	42	13.5	47	11.9	0.0001	46	12.5

## Discussion

We evaluated outcomes and costs of treating genotype 1 patients with first generation protease inhibitors TVR and BOC in a real-life setting using data from the German Hepatitis C Registry. Data on 1,786 patients was provided by 191 outpatient practices and hospital outpatient departments throughout Germany using a web-based data recording system. To our knowledge, this is the largest real-life cohort of patients receiving first generation protease inhibitors TVR or BOC so far.

We observed SVR-rates of 62.2% for TVR and 55.7% for BOC in treatment-naive patients and thus confirm results from an interim analysis with a smaller patient sample [18]. These rates are considerably lower than rates observed in clinical trials (TVR: 75%; BOC: 66%) [25;26]. Similar results were shown for treatment-experienced patients [27;28]. In total, 153 patients (8.6%) were lost to follow-up or not tested. These findings are frequently observed as clinical trial populations usually do not reflect the population treated in routine care. Furthermore, treatment monitoring is more frequent and compliance is encouraged more intensely and treatment discontinuations are less frequent due to strict study protocols. Therefore, higher rates of lost to follow-up patients are not unusual in real-life settings.

Another US study by Backus et al. (2014) evaluated outcomes of TVR and BOC treatment in a cohort of HCV-infected veterans. SVR-rates did not significantly differ between both treatments in the total cohort (TVR: 52%, BOC: 50%, p = 0.72) as well as subgroups. Outcome in treatment-naive patients were 55% and 53% for TVR and BOC, respectively. Lower SVR-rates were observed for treatment-experienced patients, except for patients with prior relapse. Further, patients with cirrhosis were less likely to achieve SVR [29].

Two other studies by Vo et al. (2015) and Wehmeyer et al. (2014) confirm these results and prove that results from clinical trials could not be easily reproduced in routine care [17;30]. Vo et al. showed SVR-rates of 53% for TVR and 40% for BOC.[17] Wehmeyer et al. observed a SVR-rate of 60.8% whereas no significant differences between TVR and BOC were observed [30]. Both analyses confirm lower SVR-rates in patients with cirrhosis.

In treatment-naive patients average treatment costs of €50,598 for TVR and €39,081 for BOC were observed. In treatment-experienced patients average treatment costs were slightly higher. We estimated average costs per SVR of €81,347 for TVR and €70,163 for BOC in treatment-naive patients. In treatment-experienced patients costs per SVR were about €80,000 in prior relapse for both TVR and BOC. In prior non-responder costs were 116,509 €/SVR and 110,156 €/SVR for TVR and BOC, respectively.

A study by Bichoupan et al. (2014) showed median treatment costs per SVR of $189,338 for patients receiving antiviral treatment with TVR in the United States [16]. These data should be interpreted with caution as 35% of patients had advanced fibrosis/cirrhosis and 73% had received dual therapy with PegIFN and RBV in the past. Median treatment costs were $83,721 and only 44% of patients achieved SVR. Subgroup analyses showed higher costs and lower SVR rates in treatment-experienced patients and patients with advanced fibrosis/cirrhosis [16].

Compared to real-life treatment outcomes and costs in genotype 1 patients receiving dual therapy with PegIFN and RBV, our analysis shows higher costs per SVR.[19] Average costs per SVR in dual treatment were €44,744 in treatment-naive patients, €73,816 in patients with prior relapse and €81,796 in patients with prior non-response. Furthermore, the decrease of QoL during treatment was greater in patients receiving TVR or BOC compared to dual treatment with PegIFN and RBV [19].

Therefore, this analysis supports the findings from previous studies and demonstrates that triple therapy with TVR or BOC is associated with a significant reduction in QoL mostly due to therapy-related side effects/adverse events. Further, we could show that the achievement of SVR is associated with an increase in QoL.

Long-term cost effectiveness of TVR and BOC treatment including costs of disease progression in patients who do not achieve SVR in respective treatments have been comprehensively evaluated in different countries (e.g. USA, Italy, UK, Germany) [31–37]. These analyses show that TVR and BOC could be considered as cost-effective. Model results are mainly influenced by the discount rate, SVR-rates and treatment costs [31–37].

A major limitation of our study is that patients were not randomly assigned to participate. Patients were recruited by study centers based on availability and their willingness to participate. This could lead to a response bias. However, potential bias could be regarded as low due to the large patient sample. Although healthcare utilisation was collected timely by filling out standardized form in the web-based recording system, missing data (e.g. medical specialist visits) might lead to an underestimation of actual costs. As major cost drivers are HCV-medication and HCV-related diagnostics, the impact of missing data is low. Results are based on a homogeneous population consisting of Caucasian patients. Therefore, transferability of results to other countries may be limited. However, the strengths of our study are comprehensive information on patient characteristics, a data quality assurance system and a large patient sample provided by a large number of study centers throughout Germany reflecting routine care in daily practice.

Recent developments in treating HCV have increased SVR-rates in all HCV genotypes, shortened treatment duration and lead to a favorable side effect profile. These new DAAs allow treating patients with prior contraindication for antiviral treatment due to interferon intolerance, advanced stages of liver disease, older age or comorbidities. In treatment-naive genotype 1 patients, treatment with sofosbuvir/ledipasvir (SOF/LDV) with or without RBV or combination treatment with paritaprevir/ritonavir/ombitasvir/dasabuvir (PTV/r/OMV/DSV) with or without RBV results in SVR-rates above 95% [38–42]. High SVR-rates were also achieved in treatment-experienced genotype 1 patients as well as in other genotypes [43;44]. These advances in HCV-treatment were accompanied by a further increase in treatment costs and have raised the question, whether such high prices are affordable [45–47]. For example, weekly costs of new agents are $8,750 for SOF/LDV, $7,000 for SOF, $6,943 for PTV/r/OMV/DSV in the United States. Due to high costs several European countries have delayed the introduction of new DAAs and restricted the use for patients with advanced liver disease [48–50]. Despite high prices, new DAA regimens SOF/LDV and PTV/r/OMV/DSV seem to be cost-effective considering national or international accepted willingness-to-pay thresholds [51–54]. Due to significant differences in health systems, structures of healthcare provision and costs between countries, transferability of economic evaluation results is limited [55]. Therefore, there is high need for national health economic evaluation results from real-life studies as well as short and long-term modelling studies.

Summarizing the results of our study, we estimated average costs of hepatitis C triple-therapy with TVR or BOC therapy in clinical practice ranging between €39,081 in treatment-naive patients receiving BOC and €53,491 in treatment-experienced patients (relapser) receiving TVR. Costs per SVR in treatment-naive patients were €81,347 and €70,163 for TVR and BOC, respectively. Highest costs per SVR were observed in patients with non-response to prior treatment (TVR: €116,509; BOC €110,156). Further, insights on QoL of HCV-infected patients were described. Therefore, these data can set a basis for a comparison with newly introduced DAA regimens and help to objectify the current discussion concerning high prices for HCV medication.
